# BiasFormer: Structured Posterior-Guided Transformer for Temporal Action Segmentation

**DOI:** 10.3390/s26154854

**Published:** 2026-08-01

**Authors:** Dongyue Zhou, Zhihui Shi, Haixia Wang, Hanqing Yang, Mingyue Yang, Chongchong Yu

**Affiliations:** School of Computer and Artificial Intelligence, Beijing Technology and Business University, Beijing 100048, China; 2306010132@st.btbu.edu.cn (D.Z.); 2431062171@st.btbu.edu.cn (Z.S.); 2306010121@st.btbu.edu.cn (H.W.); 2406080118@st.btbu.edu.cn (H.Y.); 2406080119@st.btbu.edu.cn (M.Y.)

**Keywords:** temporal action segmentation, Transformer, structured prediction, segmental CRF, action recognition

## Abstract

Transformer-based architectures have become a dominant paradigm for temporal action segmentation because of their ability to capture long-range temporal dependencies. However, content-driven self-attention does not explicitly model action durations or feasible action transitions, which can lead to short spurious fragments and ambiguous action boundaries. To address this limitation, we propose BiasFormer, a unified end-to-end framework that couples feature-level structural guidance with decoder-level structural constraints. Specifically, frame-level confidence biases derived from structured Markov posteriors guide reliability-aware feature reweighting in frame-to-action cross-attention. We further introduce the unified segmental Markov head, which formulates temporal action segmentation as a segmental conditional random field with explicit duration modeling and transition support constraints. The structured objective is jointly optimized with the backbone, enabling structural information to influence both feature interaction and decoding. Extensive experiments on Breakfast, GTEA, EgoProceL, and EPIC-KITCHENS demonstrate competitive performance and improvements over FACT on most segment-level metrics. These results support the effectiveness of coupling posterior-guided feature refinement with structured decoding for improving segment-level temporal coherence.

## 1. Introduction

Temporal action segmentation (TAS) is a fundamental task in video understanding that aims to assign an action label to each frame of an untrimmed video and thereby partition it into semantically meaningful action segments [1,2,3]. Unlike conventional action recognition, which focuses primarily on video-level classification, temporal action segmentation requires fine-grained temporal understanding: the model must not only recognize the action categories being performed but also accurately localize their temporal start and end boundaries [1,2,3].

Temporal action segmentation architectures have evolved from temporal convolutional networks (TCNs) toward Transformer-based designs [2,3,4,5,6]. Early studies mainly relied on multi-stage convolutional architectures, represented by TCNs, to capture local temporal patterns [2,3,4,5]. With the success of Transformers [7] in natural language processing and the widespread adoption of Vision Transformers (ViTs) [8] in computer vision, the advantages of self-attention-based architectures have become increasingly evident. By capturing global contextual information [7,8,9], attention-based and Transformer-inspired architectures can establish dependencies across long videos and have become influential in temporal action segmentation [6,10,11].

Transformer-based methods have become a dominant paradigm in temporal action segmentation and have achieved strong performance through designs such as cross-attention [10], hierarchical context modeling [11], and locality-aware attention [6]. In parallel, differentiable temporal logic objectives and classical structured sequence models introduce explicit temporal rules, transitions, durations, or globally feasible label sequences [12,13,14,15,16]. Despite progress in both directions, tightly coupling segment-level posterior inference with internal Transformer feature interaction under a shared training and decoding formulation remains underexplored.

Although attention-based global interaction mechanisms have achieved strong performance on several benchmarks [6,10,11,12], their direct application to action segmentation still presents limitations. Positional encoding provides absolute or relative temporal coordinates [6,7], but standard self-attention remains driven mainly by content similarity rather than temporal proximity or segment-level continuity. Consequently, attention-based models may still produce oversegmented predictions, with high-frequency label changes inside an action segment and ambiguous action boundaries [4,17,18].

To alleviate this issue, existing methods have introduced local inductive biases or temporal consistency constraints at different levels [4,6,11,16,17,18]. For instance, some approaches incorporate hierarchical context modeling, local convolutions, or locality-aware designs into the backbone to enhance the modeling of neighboring temporal relations [6,11,19]. Others improve the temporal consistency of predicted sequences at the output level through strategies such as smoothing losses, boundary modeling, or Viterbi decoding [4,13,16,17]. These approaches provide effective feature-side or output-side solutions to oversegmentation. Building on both directions, BiasFormer introduces an explicit duration and transition structure into feature interaction and jointly optimizes it with structured decoding in an end-to-end framework [14,15,16].

BiasFormer addresses this gap through a differentiable posterior feedback mechanism. The segmental model summarizes duration- and transition-aware consistency and feeds frame-level confidence into frame-to-action (F2A) cross-attention. The same structured formulation scores and decodes feasible segment sequences and is optimized jointly with the backbone. This design provides soft posterior guidance during feature interaction and explicit structural constraints during decoding.

The main contributions of this paper are summarized as follows:

1. We introduce a differentiable posterior feedback interface that converts duration- and transition-aware segmental posteriors into frame reliability biases for Transformer cross-attention.
2. We couple this soft feature-level guidance with USMH, a segmental CRF decoder, under a unified end-to-end objective, allowing one structured model to support feature interaction, structured training, and constrained decoding.
3. Extensive experiments on four temporal action segmentation benchmarks demonstrate competitive performance and improvements over the FACT backbone on most segment-level metrics; component ablations further support complementary roles for posterior guidance and structured decoding.

## 2. Related Work

### 2.1. Temporal Action Segmentation Methods

Early studies on temporal action segmentation primarily built on sequence models and segmental modeling. These works introduced temporal dependencies by explicitly modeling state transitions, action durations, or frame-wise temporal classification [1,20,21]. For example, Singh et al. proposed a multi-stream bidirectional RNN architecture for the frame-level classification of fine-grained actions [21], while Lea et al. combined convolutional features with segmental temporal modeling to address action segmentation within a unified framework [20]. These efforts laid an important foundation for subsequent research on explicit temporal structure modeling.

With the development of deep learning, temporal convolutional networks (TCNs) have become a major paradigm in this area [2,3]. By stacking dilated convolution layers, TCNs capture local temporal patterns while gradually expanding the receptive field [2]. The MS-TCN model proposed by Farha et al. adopts a multi-stage cascade to iteratively refine predictions and introduces a smoothing loss to suppress high-frequency label fluctuations [4]. MS-TCN++ redesigns the temporal blocks with dual dilated layers to combine complementary receptive fields [5]. Overall, these methods have made notable progress in modeling local temporal patterns and alleviating oversegmentation, and they have long represented an important line of work in temporal action segmentation.

Despite their effectiveness, TCN-based methods leave room for improvement in temporal structure modeling. Although dilated convolutions expand the receptive field as the depth increases, their interaction patterns remain largely determined by the convolutional architecture, and they do not adapt their dependency ranges to the input content as attention mechanisms do. Moreover, many approaches improve prediction smoothness and segmentation quality mainly through multi-stage refinement, auxiliary losses, or boundary enhancement. Injecting an explicit temporal structure earlier into feature interaction and unifying it with subsequent structured decoding therefore remains an open direction.

### 2.2. Transformer-Based Action Segmentation Models

Motivated by the success of Transformers in modeling long-range dependencies, recent studies have introduced self-attention into temporal action segmentation. Self-attention enables content-dependent interactions between distant temporal positions and captures global contextual information [7]. For long-range video understanding, Sener et al. explored temporal aggregate representations for context modeling [22], while the non-local neural network proposed by Wang et al. provided an influential basis for global dependency modeling in video tasks [9]. Yi et al. subsequently proposed ASFormer, which uses an encoder–decoder Transformer architecture to model and refine frame-wise features and achieved competitive results on multiple benchmarks [6].

Recent methods further extend temporal modeling for TAS. For example, FACT introduces a dual-branch design with frame and action branches and exchanges information through cross-attention [10]. Xu et al. incorporate differentiable temporal logic constraints into TAS training [12], while hierarchical context Transformers capture temporal dependencies at multiple resolutions [11]. Related visual sequence models have also explored frequency-aware feature mixing for skeletal action recognition [23] and spatiotemporal graph convolutions for action segmentation [24]. Frequency-domain filtering, hierarchical context aggregation, and graph message passing, respectively, target feature noise, multi-scale context, and relational structure; they do not, by themselves, provide the segmental posterior-to-attention feedback used by BiasFormer. Because frequency-aware skeletal recognition uses a different task and input modality, we include it only as a conceptual comparison rather than a numerical baseline.

In summary, Transformer-based approaches and structured sequence models offer complementary strengths: the former provide flexible long-range feature interaction, whereas the latter explicitly represent durations, transitions, and sequence-level feasibility. BiasFormer integrates these strengths by using structured posterior information to guide feature interaction and by applying the same segmental formulation to end-to-end training and decoding.

Table 1 summarizes the relations between representative temporal or structured models and BiasFormer.

### 2.3. Oversegmentation and Temporal Consistency

The tendency of models to produce oversegmentation (i.e., unreasonable high-frequency action changes) has long been a core issue in temporal action segmentation. To address this issue, prior work has proposed strategies to enhance temporal consistency from multiple perspectives. Beyond the smoothing loss in MS-TCN [4], one line of methods improves segment-level prediction quality via explicit boundary modeling. For example, the Action Segment Refinement Framework (ASRF) proposed by Ishikawa et al. introduces a boundary regression branch in parallel with the frame-wise classification branch and uses predicted boundaries to adjust the frame-level results, with reported improvements in oversegmentation-related metrics and segment-level F1 [17]. Another line of work performs temporal reasoning via graph neural networks; for instance, GTRM by Huang et al. captures long-range dependencies by constructing inter-frame graph relations and enhances sequence consistency [18]. Moreover, some approaches add extra temporal reasoning modules on top of frame-wise predictions to further improve action boundaries and label continuity.

In addition to structural enhancement within neural networks, post-processing based on sequence-level inference has a long tradition. Classic methods often introduce Markov or semi-Markov models on top of frame-wise predictions and impose transition constraints and duration priors via Viterbi decoding [13,15,16]. Such methods can explicitly restrict the label sequence so that it satisfies basic temporal consistency—for example, by avoiding rapid back-and-forth switching—thereby effectively reducing oversegmentation. However, these approaches typically apply structural constraints after the network outputs, making them closer to sequence-level correction than to direct temporal structure modeling during feature learning.

Neural CRFs, semi-Markov CRFs, and hybrid RNN-HMM models provide established foundations for differentiable or structured sequence modeling [14,15,16]. Building on these foundations, BiasFormer converts the segmental marginal posterior into a frame-wise reliability signal that directly modifies Transformer feature interaction. By sharing the structured formulation across posterior guidance, training, and decoding, BiasFormer extends the segmental structure from output inference to representation learning.

## 3. Methodology

This section describes the components required to understand BiasFormer. Detailed forward–backward recursions, Viterbi backtracking, boundary conditions, transition mask construction, and numerical implementation are provided in the Supplementary Materials.

### 3.1. Overview

**Figure 1:**
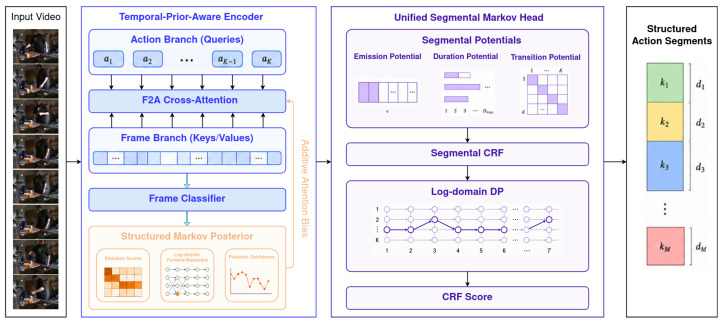
Architecture of BiasFormer during training and inference. The frame classifier provides log-emission scores; log-domain forward–backward inference combines them with duration and transition potentials to obtain frame marginals; and the log of the maximum marginal is injected through the orange feedback path as an additive F2A attention bias. During training, the posterior remains differentiable and USMH supplies the structured negative log likelihood. During inference, the same posterior-guided F2A path is followed by Viterbi decoding. The confidence bias is a class-agnostic reliability signal and does not assign class identities to action tokens. Black arrows denote the main forward processing flow, blue arrows indicate the frame-classifier-to-posterior path, and the orange arrow denotes posterior feedback to F2A; different colors in the output column represent different predicted action classes.

As shown in Figure 1, BiasFormer augments a FACT-style frame–action backbone by using a shared segmental model in two ways. First, the model converts duration- and transition-aware frame marginals into a scalar reliability bias for F2A cross-attention. Second, USMH uses the same potential family to train and decode segment sequences. The former provides a soft feature-level signal, whereas the latter constrains the decoded sequence to the configured duration bound and transition support.

The interaction proceeds in five steps. First, the frame branch produces frame-level logits, which are converted into log-emission scores. Second, log-domain forward–backward inference combines these emissions with the duration and transition potentials to compute frame marginals. Third, the logarithm of the maximum marginal at each frame forms a scalar reliability bias. Fourth, this bias is added to the F2A attention logits before the softmax, thereby reweighting the frame keys used by the action tokens. Fifth, USMH applies the segmental score to structured negative-log-likelihood training and Viterbi decoding. The posterior tensor is retained in the automatic differentiation graph rather than being detached; consequently, the frame-level auxiliary loss and the structured loss update the backbone, F2A module, and structured model parameters jointly in one backpropagation pass, without EM-style alternating optimization. During inference, the posterior-guided F2A path remains active, training losses are omitted, and max-sum dynamic programming returns the highest-scoring feasible segment sequence. This coupling distinguishes BiasFormer from applying an independent CRF only after a Transformer prediction.

### 3.2. Unified Discriminative Segmental Formulation

Given an input feature sequence $X={\left\{x_{t}\right\}}_{t=1}^{T}$, where *T* is the number of input temporal steps and $x_{t}$ is the pre-extracted video feature at time *t*, temporal action segmentation aims to predict the corresponding label sequence $Y={\left\{y_{t}\right\}}_{t=1}^{T}$. Each $y_{t}\in C$, where C is the action category set and K=|C| is the number of classes. The structured model operates on the decoder time sequence of length $T^{\prime }$; when temporal sampling changes the resolution, the labels are mapped to this same decoder time grid for structured training, and decoded labels are subsequently restored to the input length for evaluation. We convert the decoder time label sequence into $S={\left\{s_{n}\right\}}_{n=1}^{N}$, where *N* is the number of segments and each segment $s_{n}=\left(k_{n},d_{n}\right)$ consists of an action category $k_{n}\in C$ and a duration $d_{n}\in N^{+}$, satisfying $\sum_{n=1}^{N}d_{n}=T^{\prime }$.

The state $k_{n}$ denotes a semantic action category. Each dataset’s standard label mapping assigns these categories fixed model indices in {0,…,K−1}, and the transition mask is applied to these indices without additional category remapping. Accordingly, the ordered masks used here are dataset-level index-order regularizers, rather than claiming that the semantic categories possess a universal intrinsic order. Their topology and, where applicable, the maximum jump are fixed before model fitting and kept unchanged across all splits of a dataset. Mask construction uses only the number and fixed indices of the classes; it does not use transition counts from training annotations, validation or test annotations, task identity, or transcripts.

The start time $t_{n}^{s}$ and end time $t_{n}^{e}$ of the *n*-th action segment on the decoder time grid are defined as follows, where *m* indexes the preceding segments:

$$t_{n}^{s}=1+\sum_{m=1}^{n-1}d_{m},\;t_{n}^{e}=t_{n}^{s}+d_{n}-1$$

We model the conditional probability P(S∣X) with a discriminative segmental CRF, where Θ denotes all learnable parameters and Z(X) is the partition function over all candidate segment sequences satisfying $\sum_{n}d_{n}=T^{\prime }$ and the transition support constraints:

$$P\left(S|X;\Theta \right)=\frac{1}{Z(X)}\exp\left(F(X,S;\Theta )\right)$$

Here, F(X,S;Θ) is the energy (score) function defined over the input *X* and the segment sequence *S*. The energy function is decomposed into three potentials: the emission potential $\psi_{\mathrm{emit}}$, the duration potential $\psi_{\mathrm{dur}}$, and the transition potential $\psi_{\mathrm{trans}}$:

$$F\left(X,S;\Theta \right)=\sum_{n=1}^{N}\left(\psi_{\mathrm{emit}}\left(X,t_{n}^{s},t_{n}^{e},k_{n}\right)+\psi_{\mathrm{dur}}\left(d_{n},k_{n}\right)+\psi_{\mathrm{trans}}\left(k_{n-1},k_{n}\right)\right)$$

To avoid boundary condition ambiguity, we introduce a virtual start state $k_{0}=\langle \mathrm{BOS}\rangle$ and set $\psi_{\mathrm{trans}}\left(k_{0},k_{1}\right)=0$ (i.e., no transition cost for the first segment); the remaining segments use the standard adjacent-segment transition potential $\psi_{\mathrm{trans}}\left(k_{n-1},k_{n}\right)$.

The maximum candidate duration $D_{\max}$ bounds the segmental search. When $D_{\max}=1$, the segment representation reduces to one temporal step per segment; values greater than one allow explicit multi-step durations.

### 3.3. Structured Markov Posterior-Guided Feature Encoding

To address the limitations of standard Transformer architectures in explicitly modeling temporal structure, we introduce a temporal inductive bias guided by structured Markov posteriors into the backbone network. Specifically, we adopt a FACT-style frame–action dual-branch backbone consisting of a frame branch and an action branch. The frame branch extracts frame-level features, while the action branch uses action tokens to model action-level representations; the two branches exchange information bidirectionally through the frame-to-action (F2A) cross-attention module. Our method operates primarily on this interaction stage and the subsequent structured decoding stage, without changing the basic dual-branch paradigm of the backbone. We refer to this mechanism consistently as the posterior-guided confidence bias.

We leverage a structured Markov posterior with explicit duration and transition support constraints to extract frame-level structural consistency confidence, and we use this confidence to perform the lightweight reweighting of frame-level features in cross-attention. This bias does not directly specify a category-level transition topology for each action token; instead, it extracts the per-timestep structural consistency confidence from the structured Markov posterior and uses this signal to reweight frame-level features according to their reliability.

Specifically, we use the forward–backward algorithm to compute the posterior probability $\gamma_{t,k}$ that decoder step *t* belongs to action class *k*, where $t\in \{1,\ldots ,T^{\prime }\}$, k∈C, and $\sum_{k\in C}\gamma_{t,k}=1$ for each decoder step. This posterior integrates the current semantic predictions, duration constraints, allowed transition support, and temporal context. Based on this, we define the maximum posterior confidence $b_{t}$ at decoder step *t* as

$$b_{t}=\log\;\left(\max\left\{\max_{k\in C}\gamma_{t,k},\epsilon \right\}\right)$$

where ϵ∈(0,1) is a numerical floor. Because $\gamma_{t,k}\in \left[0,1\right]$, $b_{t}$ is non-positive; values closer to 0 indicate a more confident state assignment under the current structured model, whereas smaller values often correspond to boundary neighborhoods or structurally uncertain frames. We inject this bias term into the logit computation of F2A cross-attention as a soft constraint:

$${\text{attn\_logit}}_{q,i}^{\prime }=\frac{\langle K_{i},Q_{q}\rangle }{\sqrt{d_{a}}}+\lambda_{\mathrm{attn}}\cdot b_{i}$$

Here, *q* indexes action tokens, *i* indexes frames/keys, $Q_{q}\in R^{d_{a}}$ denotes the query of the *q*-th action token, $K_{i}\in R^{d_{a}}$ denotes the key of the *i*-th frame, $d_{a}$ is the attention projection dimension, and $\lambda_{\mathrm{attn}}$ is the attention bias scaling factor. Since $b_{i}$ depends only on the frame/key index *i*, the bias is broadcast along the query dimension.

This design acts more like a frame-level reliability gate induced by structured Markov posteriors than an explicit encoding of query-specific category transitions. That is, the correspondence between tokens and class semantics is still learned primarily from the content-dependent term $\langle K_{i},Q_{q}\rangle$, while the additive bias suppresses structurally unstable or posterior-uncertain frames, thereby reducing high-frequency attention jitter caused by local noise.

The scalar maximum is used as a class-agnostic reliability gate; class discrimination remains in the content-dependent dot product. Decoder time marginals are obtained by summing the posterior probabilities of all candidate segments of class *k* that cover step *t*. The posterior tensor and the resulting confidence bias are not detached from the computation graph. When temporal sampling changes the resolution, each decoder time bias value is aligned with the center index of its corresponding temporal sampling bin before F2A injection. We set $\epsilon =10^{-8}$, $\sigma_{\min}=10^{-3}$, and the finite surrogate for an unsupported transition to $-C=-10^{4}$. Full marginalization equations and implementation details are given in Supplementary Sections S1 and S2.

### 3.4. Unified Segmental Markov Head

The core task of the decoder is to find the optimal segment sequence over the log-emission sequence $E\in R^{T^{\prime }\times K}$ under structured constraints. Here, $T^{\prime }$ denotes the decoder temporal length, *K* is the number of action classes, and $E_{t,k}=\log\mathrm{Softmax}{\left(z_{t}\right)}_{k}+v_{k}$ is obtained from the classifier logits $z_{t}$ and a learnable class-specific emission bias $v_{k}$ at decoder step *t*. The corresponding potential functions are fully differentiable.

For the emission potential, we precompute prefix sums of frame-level scores, $P_{t,k}=\sum_{i=1}^{t}E_{i,k}$, with $P_{0,k}=0$, so that the accumulated score of a segment of arbitrary length can be computed efficiently. For the *n*-th candidate segment, let its decoder time interval be $\left[t_{n}^{s},t_{n}^{e}\right]$ and its class be $k_{n}$, where $1\leq t_{n}^{s}\leq t_{n}^{e}\leq T^{\prime }$. The emission potential of this segment can then be computed in O(1) time:

$$\psi_{\mathrm{emit}}\left(X,t_{n}^{s},t_{n}^{e},k_{n}\right)=P_{t_{n}^{e},k_{n}}-P_{t_{n}^{s}-1,k_{n}}$$

For the duration potential, we adopt log-space Gaussian modeling to improve the numerical stability given the long-tailed nature of action durations. For a candidate duration $d\in \{1,\ldots ,D_{\max}\}$ and class *k*, the duration potential is defined as

$$\psi_{\mathrm{dur}}\left(d,k\right)=\log N\left(\log d;\mu_{k},\sigma_{k}^{2}\right)$$

Here, $\mu_{k}$ and $\sigma_{k}$ are duration parameters learned for class *k*, $\sigma_{k}$ is constrained to be at least $\sigma_{\min}$, and logN(·) denotes the Gaussian log density in log-duration space. This potential assigns lower scores to durations that are inconsistent with the learned class-specific distribution. For the tabular duration model used on EgoProceL and EPIC-KITCHENS, the duration potential is instead a learnable lookup value $w_{k,d}$ for each class–duration pair, with $1\leq d\leq D_{\max}$.

For the transition potential, a binary mask $M\in {\left\{0,1\right\}}^{K\times K}$ defines the support set. Here, $M_{ij}=1$ permits the transition i→j, and $U_{ij}$ is its learnable score. The exact model assigns zero probability to transitions outside the support:

$$\psi_{\mathrm{trans}}\left(i,j\right)=\left\{\begin{array}{cc} U_{ij}, & M_{ij}=1\\ -\infty , & M_{ij}=0 \end{array}\right.$$

In finite-precision computation, −∞ is represented by the large negative value −C, with $C=10^{4}$. Thus, −C is the numerical surrogate for the hard transition support constraint rather than an additional learnable soft penalty.

The reported experiments use two deterministic ordered supports. For monotone_skip with maximum jump *J*, $M_{ij}=1$ if and only if 0<j−i≤J, which excludes segment-level self-transitions and backward-index transitions. For upper, $M_{ij}=1$ if and only if j≥i, allowing segment-level self-transitions and unrestricted forward jumps while excluding backward-index transitions. Under upper, $D_{\max}$ bounds each latent segment; adjacent same-class latent segments may collapse into a longer contiguous frame-level action run after decoding. Breakfast uses monotone_skip with J=3, EgoProceL and EPIC-KITCHENS use monotone_skip with J=2, and GTEA uses upper. These choices were set as dataset-level architectural regularizers before training and were not selected retrospectively from the sensitivity results. Section S3 of the Supplementary Materials gives the construction rules and provenance table.

Table 2 reports the configurations used for the main results. $D_{\max}$ is measured in decoder time steps. The transition mode and maximum jump are dataset-level configuration choices and remain fixed across splits; the later sensitivity study is diagnostic and does not alter these main result settings.

### 3.5. Optimization and Inference

During training, the forward dynamic program computes the log-partition function logZ(X) over valid segment paths. The total loss combines the structured negative log likelihood with an auxiliary frame-level cross-entropy loss. Let $S_{\mathrm{GT}}$ be the ground-truth segment sequence obtained by merging consecutive identical frame labels, and let $\lambda_{\mathrm{aux}}$ be the auxiliary loss weight:

$$L_{\mathrm{total}}=\left(\log Z\left(X\right)-F\left(X,S_{\mathrm{GT}};\Theta \right)\right)+\lambda_{\mathrm{aux}}L_{\mathrm{frame}}$$

At inference, replacing log-sum-exp aggregation with maximization yields the highest-scoring path in the configured support set. If $E_{M}=\left\{\left(i,j\right):M_{ij}=1\right\}$, the dynamic-programming cost is $O(T^{\prime }D_{\max}|E_{M}|)$ and becomes $O(T^{\prime }D_{\max}K^{2})$ for a fully connected mask. Prefix sums, forward/backward states, and Viterbi backpointers require $O(T^{\prime }K)$ dynamic programming storage when candidate scores are streamed rather than materialized. The full recursions, BOS condition, prefix-sum implementation, Viterbi backtracking, and stability constants are provided in Supplementary Sections S1 and S2. Section 4.5 compares these analytical costs with the parameter and GFLOP values reported for FACT.

## 4. Experiments

We evaluate BiasFormer for fully supervised temporal action segmentation on four benchmark datasets: Breakfast, GTEA, EgoProceL, and EPIC-KITCHENS. The evaluation includes comparisons with representative methods, component ablations, repeated-run statistics, and one-factor-at-a-time sensitivity analyses.

The experiments address the following questions:

- **(Q1)** Can BiasFormer improve segment-level performance relative to the FACT backbone? We assess this question using Edit and F1@{10,25,50} in the quantitative comparisons reported below.
- **(Q2)** Do the posterior-guided confidence bias and USMH provide individual and complementary benefits, and does end-to-end structured training yield further gains? The component ablation reported below addresses this question.

### 4.1. Datasets

BiasFormer is evaluated on both a third-person dataset (Breakfast) and egocentric datasets (GTEA, EgoProceL, and EPIC-KITCHENS), covering diverse capture conditions, action granularities, and sequence lengths.

Breakfast Actions Dataset. Breakfast contains long breakfast preparation videos captured by multiple cameras in real kitchen environments and is one of the larger fully annotated benchmarks for temporal action segmentation. It includes approximately 77 h of video (more than four million frames) from 52 participants across 18 kitchens, with 48 coarse action classes [1,25]. In the action segmentation setting, the task is to detect and classify fine-grained action units along the temporal axis.

GeorgiaTech Egocentric Activities (GTEA). GTEA is an egocentric dataset recorded by head-mounted cameras during daily activities. The temporal action segmentation benchmark contains 28 videos from four subjects and 11 fine-grained action classes [10,26]. We follow the standard four-fold leave-one-subject-out protocol used in prior action segmentation work.

EgoProceL. EgoProceL is a large-scale egocentric dataset for procedural learning. It contains 62 h of video from 130 subjects performing 16 tasks [27]. The FACT action segmentation protocol uses 1055 videos and 130 annotated action or key step classes [10]. We follow this protocol and treat the step annotations as frame-wise labels for segmentation evaluation.

EPIC-KITCHENS [28]. EPIC-KITCHENS is one of the largest egocentric vision datasets and contains natural daily activities performed in participants’ own kitchens. We follow the EK-100 evaluation protocol; the source dataset includes 100 h of video, approximately 20 million frames, and 89,977 action segments across 700 variable-length videos recorded in 45 kitchens. Its diverse viewpoints and rich verb–noun action labels make it particularly challenging.

For readability, Table 3 summarizes the datasets and evaluation protocols.

#### Data Split and Subject Independence Protocol

Subject overlap and sample leakage are related but distinct concerns. Reusing an identical video or derived frame sequence across training and test sets is direct sample leakage. Using different videos from the same participant does not duplicate samples, but it can still produce an optimistic estimate of performance for unseen participants because subject-specific visual and behavioral cues may be shared [29]. No random frame-level partitioning is used in our experiments: every complete video sequence is assigned to a single split, and the released train and evaluation video lists are disjoint. We therefore distinguish video disjointness from participant disjointness and interpret each benchmark only within its established protocol.

Breakfast uses the official four-fold participant-based split, and GTEA uses the standard four-fold leave-one-subject-out protocol; both evaluations are participant-disjoint within each fold. EgoProceL uses the 80/20 complete-video split released with FACT. This split is video-disjoint but was not designed as a participant-independent protocol, so its results characterize held-out video performance rather than generalization exclusively to unseen participants. EPIC-KITCHENS uses the official EK-100 split, whose evaluation data include both seen- and unseen-participant conditions; the aggregate results are therefore interpreted under the official mixed-participant protocol rather than as a purely participant-independent evaluation. Table 4 summarizes these protocol sources and interpretation scopes.

### 4.2. Experimental Setup and Training

#### 4.2.1. Input Features and Preprocessing

We use pre-extracted I3D feature sequences for all four datasets. The feature files are taken directly from the corresponding official or FACT-released preprocessing resources or generated with the data processing pipeline provided by FACT. The resulting feature inputs used by BiasFormer are identical to those used by FACT, including the dataset splits, temporal preprocessing, and sampling settings. Feature extraction is performed before temporal model training and is not fine-tuned as part of BiasFormer.

Breakfast and GTEA use a temporal sampling rate of 1, EgoProceL uses 3, and EPIC-KITCHENS uses 4. During testing, predictions are restored to the original sequence length before evaluation, following the FACT evaluation pipeline.

#### 4.2.2. Compared Methods

We compare BiasFormer with representative TCN-based and Transformer-based temporal action segmentation methods, including

- MS-TCN++ [5] (multi-stage temporal convolution with progressive refinement);
- ASRF [17] (explicit boundary modeling and refinement);
- ASFormer [6] (Transformer with local inductive bias);
- LTContext [11] (hierarchical context Transformer capturing multi-scale temporal dependencies);
- FACT [10] (frame–action cross-attention temporal modeling, also used as the backbone in our implementation).

The comparison tables combine the results obtained from the cited publications under their established benchmark protocols with a feature-aligned comparison between FACT and BiasFormer. BiasFormer uses the same feature inputs, temporal preprocessing, sampling settings, and evaluation protocol as FACT. The remaining methods provide broader context but are not described as controlled reproductions because their feature extractors and implementation details may differ. Methods may also include distinct refinement stages or boundary modules, such as MS-TCN++ and ASRF.

#### 4.2.3. Training Configuration

BiasFormer is implemented in PyTorch and trained end-to-end. We follow the training setup of the FACT backbone and incorporate the proposed posterior-guided confidence bias and unified segmental Markov head (USMH).

The principal optimization and initialization settings used in the experiments are summarized in Table 5. The same backbone dropout rate is used in the temporal convolution, attention/feedforward, and frame–action mapping layers within each dataset configuration.

All runs use Adam with a base learning rate of $10^{-4}$, zero weight decay, no warm-up, full FP32 precision, one optimizer update per mini-batch, and no gradient accumulation. We set the auxiliary frame loss weight to $\lambda_{\mathrm{aux}}=0.2$, the USMH emission head dropout to 0, the gradient clipping threshold to 10, the unsupported transition surrogate to $C=10^{4}$, the posterior floor to $\epsilon =10^{-8}$, and the duration-scale floor to $\sigma_{\min}=10^{-3}$. For Breakfast and GTEA, the normal duration parameters are initialized as $\mu_{k}=20$ and $\sigma_{k}=10$; for EgoProceL and EPIC-KITCHENS, the tabular duration logits are initialized to zero. The FACT backbone is randomly initialized and trained from scratch, action queries are sampled from N(0,1), and transition residual scores $U_{ij}$ and emission state biases are initialized to zero; other neural layers use PyTorch defaults. Breakfast and GTEA use seeds 1, 42, and 1337, whereas EgoProceL and EPIC-KITCHENS use one training run each. For every fixed-length run, we evaluate the checkpoint with the minimum total training loss; no early stopping or validation/test-based checkpoint selection is used. The complete reproducibility record is provided in Table S2 of the Supplementary Materials.

The transition supports in Table 2 are deterministic functions of the standard model class indices. They are selected once at the dataset level as coarse temporal regularizers and are applied unchanged to every split and sample. No transition frequency estimate, validation/test annotation, task identity, or transcript is used to construct or select a mask. We do not interpret these index-based supports as exact task grammars: they deliberately trade flexibility for a stronger non-decreasing temporal prior, and valid backward-index or long-jump transitions may consequently be excluded. This limitation is evaluated separately in the later diagnostic transition-support comparison.

### 4.3. Testing Protocol

During testing, we do not use any ground-truth labels or transcripts. Given an input feature sequence, the backbone outputs frame-level logits, and USMH then performs structured inference. Specifically, we replace log-sum-exp aggregation with maximization in the forward recursion of the segmental CRF to perform Viterbi decoding, obtaining the globally optimal segmentation path within the configured transition support and duration bound.

We use the unmodified dataset-specific evaluation pipeline released with FACT [10], including its label mappings, background class handling, restoration of temporally sampled predictions to the original sequence length, and metric implementation. The reported metrics are the frame-wise accuracy excluding background frames (Acc), frame-wise accuracy including background frames (AccB, reported for EgoProceL and EPIC-KITCHENS), normalized edit score (Edit), and segment-level F1 at intersection-over-union thresholds of 0.10, 0.25, and 0.50. Higher values are better for all metrics.

### 4.4. Experimental Results

#### 4.4.1. Comparison with Representative Methods

Table 6 and Table 7 report quantitative comparisons of BiasFormer on Breakfast, GTEA, EgoProceL, and EPIC-KITCHENS. The results follow the established evaluation protocols and scripts for each dataset.

#### 4.4.2. Result Analysis

FACT [10] is the most relevant direct comparison because it supplies the frame–action backbone used by BiasFormer. BiasFormer improves most segment-level metrics over FACT across all four datasets. On Breakfast, F1@25 and F1@50 increase by 3.1 and 2.7 points, respectively; on GTEA, F1@50 increases by 2.4 points. On EgoProceL, Edit and Acc increase by 3.1 and 3.4 points, while, on EPIC-KITCHENS, F1@25 and F1@50 increase by 3.2 and 2.4 points. The gains are particularly visible under stricter segment overlap criteria, matching the structural focus of BiasFormer.

F1@50 requires correct-class predictions to satisfy a stricter overlap criterion, so improvements indicate better segment localization under the benchmark protocol. On GTEA, Edit decreases from 91.4 to 91.1 even as all F1 scores and Acc improve, showing that the benefit is not uniform across sequence metrics. F1 and Edit are aggregate measures and do not isolate a boundary or fragmentation mechanism. Accordingly, the practical interpretation is limited to benchmark segmentation quality; downstream utility, real-time deployment, and user-facing outcomes are not evaluated.

The component ablation below separates the contributions of posterior-guided attention, structured decoding, and end-to-end structured training. Multi-seed results on Breakfast and GTEA characterize run-to-run variability.

#### 4.4.3. Component Ablation on the GTEA Dataset

To examine the contributions of each component, we compare GTEA variants that isolate (i) posterior-guided confidence bias, (ii) USMH decoding, (iii) end-to-end USMH training, and (iv) their combination in the full BiasFormer model, all relative to the FACT backbone.

Table 8 shows how the individual components and their combination affect the three-seed means. Relative to FACT, the bias-only variant improves the mean F1@50 by 0.8 points, decoding-only USMH improves it by 1.3 points, and end-to-end USMH improves it by 1.9 points. Combining posterior guidance with end-to-end USMH yields the strongest mean F1@50 and Acc, improving them by 2.4 and 1.4 points, respectively. The mean improvements are consistent with the complementary roles for feature-level reliability guidance and segment-level structured learning. Across the four BiasFormer variants, the sample standard deviations range from 0.3 to 1.6 points. The end-to-end USMH variant has the largest observed variability on F1@10 (1.6 points) and F1@50 (1.0 point), while the full model remains within 0.4–0.7 points across all five metrics. Edit remains slightly below the reported FACT value for the structured variants, so the component effects are not uniformly positive across every metric.

### 4.5. Additional Analyses

#### 4.5.1. Statistical Stability

The repeated Breakfast and GTEA experiments each comprise three independently trained seeds (1, 42, and 1337). For each seed, the standard-fold results are first averaged, after which the mean and sample standard deviation are computed across seeds. Table 9 reports the verified cross-seed statistics used in the main comparison. The standard deviations range from 0.2 to 1.0 points on Breakfast and from 0.4 to 0.7 points on GTEA, indicating limited run-to-run variation under both protocols. EgoProceL and EPIC-KITCHENS use one training run per dataset, so no optimization seed stability claim is made for these datasets. A video-level bootstrap interval would characterize a different source of uncertainty and is not used as a substitute for optimization seed variation.

#### 4.5.2. Analytical Computational Cost

FACT reports 1.9 million parameters and 5.5 inference GFLOPs for a five-minute video under the comparison protocol in its original paper [10]. These are published reference values rather than measurements from our hardware. BiasFormer retains the FACT backbone and adds transition scores, one emission-state bias per class, and duration parameters. With dense K×K transition score allocation and the normal duration model used on GTEA, the additional parameter count is at most

$$\Delta P_{\mathrm{normal}}=K^{2}+3K,$$

where $K^{2}$ accounts for transition scores, *K* for emission-state biases, and 2K for the duration mean and scale. For GTEA, K=11, so $\Delta P_{\mathrm{normal}}\leq 154$, which is below 0.01% of the rounded 1.9-million-parameter FACT reference. If only supported transition entries are instantiated, the increment is smaller. For a tabular duration model, the corresponding upper bound is $\Delta P_{\mathrm{tabular}}=K^{2}+K+KD_{\max}$.

The analytical comparison shows that the structured component increases the trainable parameter count by less than 0.01% relative to the rounded FACT reference. Its dominant additional computation scales linearly with the decoder length, maximum duration, and number of supported transition edges. For GTEA, $|E_{M}|=K\left(K+1\right)/2=66$, yielding at most $13,200T^{\prime }$ transition–duration candidate evaluations per dynamic programming pass. Because GFLOPs accounting for log-sum-exp and dynamic programming kernels is implementation-dependent, Table 10 does not convert this operation count into an estimated wall-clock time or memory consumption.

#### 4.5.3. Hyperparameter and Transition Support Sensitivity

The sensitivity study is conducted diagnostically on GTEA split 1 with seed 1337 and all non-varied settings held at their main configuration values ($\lambda_{\mathrm{attn}}=1.0$, $D_{\max}=200$, and upper). Each fixed-length run uses the checkpoint with the minimum total training loss. This single-split, single-seed analysis is not used to select or revise the configurations reported in Table 2 and Table 5, and it is interpreted as a diagnostic comparison rather than a statistical stability result. Table 11 reports the resulting one-factor-at-a-time comparison.

The intermediate attention bias weight $\lambda_{\mathrm{attn}}=1.0$ gives the highest F1@50 and Acc, while 0.5 gives a 0.1-point higher Edit score. Removing the attention bias ($\lambda_{\mathrm{attn}}=0$) reduces F1@50 by 0.8 points relative to the main setting, whereas increasing the weight to 2.0 reduces it by 0.5 points. For duration, $D_{\max}=200$ gives the highest F1@50 and Acc; increasing it to 300 improves Edit by 0.2 points but lowers F1@50 and Acc by 0.2 points each. Among transition supports, upper gives the highest F1@50 and Acc, whereas free gives a 0.2-point higher Edit score. These metric-level trade-offs show limited variation across the tested settings under this diagnostic protocol, without implying that one setting dominates every criterion.

#### 4.5.4. Environment Details

The experiments were conducted in the following hardware and software environment.

**Hardware**

- CPU: Intel(R) Xeon(R) CPU E5-2698B v3 @ 2.00GHz (dual-socket, 32 cores/64 threads total)
- Memory: 256 GB DDR3
- GPU: 2 × NVIDIA Tesla V100-SXM2-16GB + 1 × NVIDIA GeForce GTX 1080 Ti

**Software**

- OS: Ubuntu 22.04.5 LTS (Jammy Jellyfish)
- Kernel: Linux 6.8.0-94-generic
- NVIDIA driver: 550.144.03
- CUDA: 12.4
- Python: 3.11.13
- PyTorch: 2.2.2
- NumPy: 1.26.4

## 5. Conclusions

BiasFormer connects segmental inference with Transformer feature interaction for structured temporal action segmentation. A duration- and transition-aware marginal posterior supplies a frame-wise reliability bias to F2A attention, while USMH uses the same segmental score for structured training and decoding. This posterior feedback coupling allows one structured model to guide both representation learning and sequence prediction.

Across Breakfast, GTEA, EgoProceL, and EPIC-KITCHENS, BiasFormer demonstrates competitive performance and improves upon FACT on most segment-level metrics, with particularly clear gains at stricter F1 thresholds. On GTEA, all F1 scores and Acc improve despite a slight decrease in Edit, indicating improved segment overlap metrics alongside a sequence–edit trade-off. Together with the component ablations, these results support the effectiveness of coupling posterior-guided feature refinement with structured training and decoding. The repeated-run analysis in Section 4.5 further characterizes the run-to-run variation on Breakfast and GTEA.

### 5.1. Limitations and Discussion

Several limitations define the scope of these results.

#### 5.1.1. Computational Cost

USMH adds dynamic programming. For the allowed transition-edge set $E_{M}$, its asymptotic cost is

$$O(T^{\prime }D_{\max}|E_{M}|).$$

The practical overhead depends on the sequence length, duration truncation, transition support density, and implementation. Table 10 therefore reports only published FACT reference values and analytical BiasFormer increments; it does not establish measured wall-clock latencies or peak GPU memory.

#### 5.1.2. Hyperparameters and Topology

$D_{\max}$, $\lambda_{\mathrm{attn}}$, and the transition support affect both accuracy and cost. The ordered supports operate on fixed model class indices and should be understood as heuristic regularizers, not exact semantic task grammars. They can exclude valid backward-index transitions or long jumps, particularly in unconstrained activities. Under upper, adjacent same-class latent segments can merge into a longer frame-level run, so $D_{\max}$ bounds each latent segment rather than the final merged run. We therefore disclose the deterministic support construction and fixed dataset-level settings and evaluate representative alternatives in Table 11.

#### 5.1.3. Evaluation Scope

Breakfast and GTEA use participant-based protocols, whereas the EgoProceL 80/20 video split and the official EPIC-KITCHENS protocol are not automatically participant-disjoint. Video disjointness prevents duplicate-video leakage but does not establish generalization to unseen participants. Table 4 makes this distinction explicit so that the results are interpreted within the scope of each benchmark protocol.

#### 5.1.4. Statistical Evidence

The repeated-run results quantify optimization seed variation on Breakfast and GTEA. Since no hypothesis test is performed and FACT is represented by values reported in its original paper, the observed differences are not described as statistically significant.

#### 5.1.5. Feature Dependency

BiasFormer depends on the quality of the features supplied to the temporal backbone. The current experiments use pre-extracted I3D features and do not evaluate end-to-end video feature learning. If the low-level feature encoder does not capture discriminative semantic information, higher-level temporal structure constraints may not fully compensate for this deficiency.

Future work will explore adaptive transition support learning, more efficient segmental inference, and subject-independent transfer. Extending the framework to weak supervision, multimodal observations, and causal streaming inference also offers promising directions.

## Figures and Tables

**Table 1:** Conceptual comparison with representative temporal and structured sequence models. The table identifies the primary relation of each method family to BiasFormer; it is not a cross-dataset performance comparison. Frequency-aware mixed Transformers operate on skeletal action recognition and are included only to clarify the difference between frequency-guided feature enhancement and segmental posterior feedback.

Representative Method	Primary Mechanism	Relation to BiasFormer
ASFormer [6]	Locality-aware Transformer with iterative refinement	Models local and long-range context without an explicit segmental posterior-to-attention interface.
FACT [10]	Frame–action cross-attention	Supplies the dual-branch backbone on which BiasFormer adds structured reliability guidance and decoding.
Hierarchical context Transformer [11]	Multi-resolution temporal context	Aggregates context across temporal scales; complementary to explicit duration and transition scoring.
Frequency-aware mixed Transformer [23]	Frequency-guided feature enhancement	Suppresses or mixes frequency components in a different action recognition setting; it does not impose a segmental feasible set.
Graph temporal models [18,24]	Graph-based temporal or spatiotemporal relation reasoning	Encode relational structure through message passing rather than segmental duration and transition potentials.
CRF and semi-Markov CRF [14,15]	Globally normalized sequence or segment scoring	Provide the structured prediction foundation; BiasFormer adds posterior feedback to Transformer feature interaction.
RNN-HMM [16]	Neural frame model with HMM alignment and decoding	Couples neural scores with Markov inference but does not use the posterior as an F2A reliability bias.
BiasFormer	Posterior-guided F2A attention plus USMH	Uses one segmental model for soft feature guidance, structured training, and constrained decoding.

**Table 2:** Dataset-specific BiasFormer configurations used for the reported main results. These fixed values are distinct from those examined in the later diagnostic sensitivity analysis.

Hyperparameter	Breakfast	GTEA	EgoProceL	EPIC-KITCHENS
$D_{\max}$	60	200	32	32
$\lambda_{\mathrm{attn}}$	0.2	1.0	0.2	0.2
transition_mode	monotone_skip	upper	monotone_skip	monotone_skip
max_jump	3	Not applicable	2	2
duration_type	normal	normal	tabular	tabular
duration_init_mu	20.0	20.0	Not applicable	Not applicable
duration_init_sigma	10.0	10.0	Not applicable	Not applicable

**Table 3:** Overview of the four benchmark datasets used for temporal action segmentation. Durations and counts describe the source datasets; the split protocol and subject independence audit are reported separately below.

Dataset	Viewpoint	Annotation Granularity	Notes
Breakfast	Third-person	Fine-grained units	∼77 h, 15 fps, standard protocol
GTEA	Egocentric	Fine-grained actions	28 videos, 4 subjects, 11 actions
EgoProceL	Egocentric	Key steps	62 h, 130 subjects, 16 tasks
EPIC-KITCHENS (EK-100)	Egocentric	Actions	100 h, 700 videos, 89,977 actions

**Table 4:** Split sources and participant independence scope. All splits are taken directly from the corresponding dataset release or the FACT experimental protocol, and no complete video appears in both training and evaluation. Video disjointness does not by itself imply evaluation on unseen participants.

Dataset	Split Source and Unit	Video Separation	Participant Scope
Breakfast	Official four-fold participant-based split [1]	Disjoint within each fold	Participant-disjoint
GTEA	Standard four-fold leave-one-subject-out split [26]	Disjoint within each fold	Participant-disjoint
EgoProceL	FACT-released 80/20 complete-video split [10,27]	Disjoint train/test video lists	Video-level evaluation; participant independence is not assumed
EPIC-KITCHENS-100	Official EK-100 video-level split [28]	Disjoint train/evaluation video lists	Official mixed seen/unseen participant evaluation

**Table 5:** Dataset-specific optimization and preprocessing settings for BiasFormer. The fixed structural settings are reported in Table 2; global optimization and initialization settings are given below.

Setting	Breakfast	GTEA	EgoProceL	EPIC-KITCHENS
Epochs	100	400	350	200
Batch size	2	1	1	1
Temporal sample rate	1	1	3	4
Backbone dropout	0.0	0.2	0.0	0.0
Channel masking probability	0.3	0.5	0.3	0.3
Step decay epoch	50	250	250	Disabled

**Table 6:** Temporal action segmentation results on Breakfast and GTEA. F1@10/25/50 are segment-level F1 scores at the indicated IoU thresholds; Edit is the normalized sequence edit score; Acc is frame-wise accuracy. All values are percentages, and higher is better. A dash denotes an unreported value, and boldface identifies the BiasFormer row. Baseline values are transcribed from the comparison under the non-transcript setting in FACT [10]; citations in the Method column identify the original methods. BiasFormer uses the same feature inputs, dataset splits, temporal preprocessing, and unmodified evaluation pipeline as FACT. The reported BiasFormer values on both datasets are means over independently trained runs with seeds 1, 42, and 1337.

	Breakfast					GTEA				
Method	F1@10	F1@25	F1@50	Edit	Acc	F1@10	F1@25	F1@50	Edit	Acc
ED-TCN [3]	–	–	–	–	–	72.2	69.3	56.0	64.0	–
TDRN [30]	–	–	–	–	–	79.2	74.4	62.7	74.1	70.1
SSA-GAN [31]	–	–	–	–	–	80.6	79.1	74.2	76.0	74.4
Bridge-Prompt [32]	–	–	–	–	–	94.1	92.0	83.0	91.6	81.2
MS-TCN [4]	52.6	48.1	37.9	61.7	63.3	87.5	85.4	74.6	81.4	79.2
MS-TCN++ [5]	64.1	58.6	45.9	64.9	67.6	88.8	85.7	76.0	83.5	80.1
MuCon [33]	73.2	66.1	48.4	76.3	62.8	–	–	–	–	–
C2F-TCN [34]	72.2	68.7	57.6	69.6	76.0	84.3	81.8	72.6	76.4	84.9
ASRF [17]	74.3	68.9	56.1	72.4	67.6	89.4	87.8	79.8	83.7	77.3
HASR [19]	74.7	69.5	57.0	71.9	69.4	90.9	88.6	76.4	87.5	77.4
ASFormer [6]	76.0	70.6	57.4	75.0	73.5	90.1	88.8	79.2	84.6	79.7
DTL [12]	78.8	74.5	62.9	77.7	75.8	–	–	–	–	–
MVGA [35]	75.6	72.1	59.7	76.8	74.2	91.3	90.0	79.3	86.4	80.3
TCTr [36]	76.6	71.1	58.5	76.1	77.5	91.3	90.1	80.0	87.9	81.1
UVAST [37]	76.9	71.5	58.0	77.1	69.7	92.7	91.3	81.0	92.1	80.2
RTK [38]	76.9	72.4	60.5	76.1	73.3	91.2	90.6	83.4	87.9	80.3
DiffAct [39]	80.3	75.9	64.6	78.4	75.1	92.5	91.5	84.7	89.6	82.2
FACT [10]	81.4	76.5	66.2	79.7	76.2	93.5	92.1	84.1	91.4	86.1
**BiasFormer (Ours)**	**82.7**	**79.6**	**68.9**	**81.0**	**79.1**	**94.1**	**93.2**	**86.5**	**91.1**	**87.5**

**Table 7:** Temporal action segmentation results on EgoProceL and EPIC-KITCHENS. F1@10/25/50 and Edit are defined as in Table 6; Acc is frame-wise accuracy excluding background frames, whereas AccB includes background frames, following FACT [10]. All values are percentages, and higher is better; boldface identifies the BiasFormer row. Baseline values are transcribed from the FACT comparison; citations in the Method column identify the original methods. BiasFormer uses the same feature inputs, dataset splits, temporal preprocessing, and unmodified evaluation pipeline as FACT.

	EgoProceL						EPIC-KITCHENS					
Method	F1@10	F1@25	F1@50	Edit	Acc	AccB	F1@10	F1@25	F1@50	Edit	Acc	AccB
MS-TCN++ [5]	60.3	57.0	46.5	62.4	69.3	82.5	15.2	13.6	9.5	11.6	18.2	27.0
ASFormer [6]	63.3	60.9	51.0	64.9	71.1	84.9	16.4	14.8	10.5	12.6	19.1	26.9
UVAST [37]	60.5	58.3	46.6	67.7	67.8	83.2	10.8	8.0	4.3	4.8	25.2	15.8
LTContext [40]	64.2	61.3	51.2	61.3	70.3	84.7	19.5	17.1	11.5	16.7	20.2	31.9
DiffAct [39]	67.5	65.4	54.6	68.4	77.0	86.6	12.5	10.9	7.2	9.6	17.1	30.9
FACT [10]	73.0	69.8	60.8	75.7	77.6	88.0	27.6	25.3	18.5	22.3	20.8	34.7
**BiasFormer (Ours)**	**75.3**	**71.2**	**61.1**	**78.8**	**81.0**	**89.6**	**29.6**	**28.5**	**20.9**	**23.7**	**21.9**	**36.1**

**Table 8:** Component ablation on GTEA. “Bias” denotes posterior-guided reliability reweighting in F2A attention; “USMH” denotes segmental structured decoding; and “Structured NLL” denotes joint training with the segmental negative log likelihood. A check mark indicates that a component is enabled, whereas a cross indicates that it is disabled. “Decoding only” applies USMH without the structured training loss. BiasFormer variants report the mean ± sample standard deviation over independently trained runs with seeds 1, 42, and 1337. For each run, the checkpoint with the minimum total training loss is evaluated. The FACT row is quoted from the cited publication and is therefore shown without a seed standard deviation. Values are percentages, and higher is better; boldface marks the largest central value in each metric column.

Variant	Bias	USMH	Structured NLL	F1@10	F1@25	F1@50	Edit	Acc
FACT (baseline) [10]	×	×	×	93.5	92.1	84.1	91.4	86.1
+ Bias only	✓	×	×	93.8±0.6	92.5±0.9	84.9±0.4	91.3±0.3	86.7±0.7
+ USMH (decoding only)	×	✓	×	93.7±1.1	92.6±0.5	85.4±0.7	91.0±0.7	86.4±0.8
+ USMH (end-to-end)	×	✓	✓	93.9±1.6	92.9±0.9	86.0±1.0	91.2±0.3	87.0±0.8
BiasFormer (full)	✓	✓	✓	94.1±0.5	93.2±0.7	86.5±0.4	91.1±0.4	87.5±0.5

**Table 9:** Direct FACT–BiasFormer comparison and multi-seed stability record on Breakfast and GTEA. The FACT values are quoted from the cited publication and are not accompanied by a seed standard deviation. BiasFormer values are the mean ± sample standard deviation over independently trained runs with seeds 1, 42, and 1337 after fold-level aggregation. All values are percentages, and higher is better.

Dataset	Method	F1@10	F1@25	F1@50	Edit	Acc
Breakfast	FACT (reported) [10]	81.4	76.5	66.2	79.7	76.2
Breakfast	BiasFormer (3 seeds)	82.7 ± 0.6	79.6 ± 0.2	68.9 ± 0.8	81.0 ± 0.6	79.1 ± 1.0
GTEA	FACT (reported) [10]	93.5	92.1	84.1	91.4	86.1
GTEA	BiasFormer (3 seeds)	94.1 ± 0.5	93.2 ± 0.7	86.5 ± 0.4	91.1 ± 0.4	87.5 ± 0.5

**Table 10:** Provenance-separated computational comparison. FACT parameter and GFLOP values are reported by Lu and Elhamifar [10] for a five-minute video and are not local measurements. BiasFormer entries are analytical increments for the GTEA configuration (K=11, $D_{\max}=200$, and upper, for which $|E_{M}|=66$). The asymptotic terms are not converted into estimated latencies or GPU memory.

Method	Trainable Parameters	Inference Computation	Additional DP State Storage
FACT	1.9 M (reported)	5.5 GFLOPs/5 min video (reported)	Not reported
BiasFormer (GTEA)	FACT +≤154	FACT $+\;O(T^{\prime }\;\times 200\times 66)$	$O(11T^{\prime })$

**Table 11:** One-factor-at-a-time sensitivity analysis on GTEA split 1 with seed 1337. The main configuration is $\lambda_{\mathrm{attn}}=1.0$, $D_{\max}=200$, and upper; all non-varied settings remain fixed at these values. Checkpoints are selected by minimum total training loss. Values are percentages, and higher is better; boldface marks the highest value within each factor block.

Factor	Setting	F1@50	Edit	Acc
$\lambda_{\mathrm{attn}}$	0	85.6	91.0	86.8
$\lambda_{\mathrm{attn}}$	0.5	86.1	**91.4**	87.1
$\lambda_{\mathrm{attn}}$	1.0 (main)	**86.4**	91.3	**87.4**
$\lambda_{\mathrm{attn}}$	2.0	85.9	91.0	87.2
$D_{\max}$	100	85.7	91.0	86.9
$D_{\max}$	200 (main)	**86.4**	91.3	**87.4**
$D_{\max}$	300	86.2	**91.5**	87.2
Transition support	free	86.1	**91.5**	87.1
Transition support	monotone_skip (J=2)	85.7	90.9	86.9
Transition support	upper (main)	**86.4**	91.3	**87.4**

## Data Availability

The datasets analyzed in this study are publicly available benchmark datasets. Implementation details and data processing protocols are described in the manuscript.

## Supplementary Materials

The following supporting information can be downloaded at https://www.mdpi.com/article/10.3390/s26154854/s1. Supplementary File S1: Detailed segmental inference and transition-support construction (Sections S1–S3, including Table S1), the split-specific reproducibility record (Section S4 and Table S2), and additional analytical complexity and sensitivity details (Section S5).

## Abbreviations

The following abbreviations are used in this manuscript:

TAS	Temporal action segmentation
TCN	Temporal convolutional network
CRF	Conditional random field
USMH	Unified segmental Markov head
F2A	Frame-to-action
