# Supplementary material for: BiasFormer: Structured Posterior-Guided Transformer for Temporal Action Segmentation
Source: Sensors (Basel). 2026 Aug 1;26(15):4854. doi: 10.3390/s26154854 (PMC13468761; doi:10.3390/s26154854)
Supplement: Supplementary file 1 [file sensors-26-04854-s001.zip › sensors-4419003-supplementary/Definitions/logo-updates-eps-converted-to.pdf]

Check for updates
