# Supplementary material for: BiasFormer: Structured Posterior-Guided Transformer for Temporal Action Segmentation
Source: Sensors (Basel). 2026 Aug 1;26(15):4854. doi: 10.3390/s26154854 (PMC13468761; doi:10.3390/s26154854)
Supplement: Supplementary file 1 [file sensors-26-04854-s001.zip › sensors-4419003-supplementary/sensors-4419003-supplementary.pdf]

Supplementary Materials: BiasFormer: Structured Posterior-Guided Transformer for Temporal Action Segmentation

Dongyue Zhou , Zhihui Shi , Haixia Wang, Hanqing Yang, Mingyue Yang and Chongchong Yu *

These supplementary materials provide detailed segmental-inference derivations, transition-support construction procedures, reproducibility specifications, and additional experimental analyses for BiasFormer.

S1. Segmental Forward–Backward Inference

Let T' be the decoder temporal length, let $z_{t,k}$ be the classifier logit for class k at decoder step t, and define the log-emission score $E_{t,k} = \log \mathrm{Softmax}(z_t)_k + v_k$, where v_k is a learnable class-specific emission bias. The emission score of a class-k segment starting at decoder index s with duration d is $\mathcal{E}(s,d,k) = \sum_{u=s}^{s+d-1} E_{u,k}$. Let $h(d,k)$ be its duration score, $a(i,k)$ its transition score from the preceding class i, and $D_{\max}$ the maximum candidate duration. For the normal model, $h(d,k)$ is the log-density of $\log d$ under the class-specific Gaussian with scale bounded below by $\sigma_{\min}$; for the tabular model, $h(d,k) = w_{k,d}$ is a learnable class–duration lookup value. A virtual beginning-of-sequence state $\langle\mathrm{BOS}\rangle$ has score zero at time 0.

The forward value $\alpha_t(k)$ is the log-sum-exp score of all valid paths that cover the first t steps and end in class k:

$$\alpha_t(k) = \mathrm{LSE}_{1\le d\le \min(D_{\max},t)}\left[\mathcal{E}(t-d+1,d,k) + h(d,k) + \mathrm{LSE}_i\{\alpha_{t-d}(i) + a(i,k)\}\right]. \tag{S1}$$

For $t-d=0$, the inner predecessor is $\langle\mathrm{BOS}\rangle$ and $a(\langle\mathrm{BOS}\rangle,k) = 0$. Other real-class states at time 0 are invalid. The log-partition function is $\log \mathcal{Z}(X) = \mathrm{LSE}_k\, \alpha_{T'}(k)$.

Define $\beta_t(i)$ as the log-sum-exp score of suffixes beginning after time t, given that the preceding segment has class i:

$$\beta_t(i) = \mathrm{LSE}_{\substack{1\le d\le \min(D_{\max},T'-t)\\ k}}\left[a(i,k) + h(d,k) + \mathcal{E}(t+1,d,k) + \beta_{t+d}(k)\right], \tag{S2}$$

with $\beta_{T'}(i) = 0$. The log posterior of a candidate segment (s,d,k) is

$$\log p(s,d,k \mid X) = \mathrm{LSE}_i\{\alpha_{s-1}(i) + a(i,k)\} + h(d,k) + \mathcal{E}(s,d,k) + \beta_{s+d-1}(k) - \log \mathcal{Z}(X), \tag{S3}$$

where the BOS convention is used when $s=1$. The decoder-step marginal is obtained by summing all candidate-segment posteriors that cover that step:

$$\gamma_{t,k} = \sum_{s,d:\ s\le t\le s+d-1} p(s,d,k \mid X). \tag{S4}$$

The class-agnostic confidence bias used by F2A is

$$b_t = \log\left(\max\left\{\max_k \gamma_{t,k}, \epsilon\right\}\right), \tag{S5}$$

where $\epsilon \in (0,1)$ is a numerical floor.

All recursions are evaluated in the log domain. Segment emissions can be queried in constant time by prefix sums $P_{t,k} = \sum_{u=1}^{t} E_{u,k}$, so that $\mathcal{E}(s,d,k) = P_{s+d-1,k} - P_{s-1,k}$.

When temporal sampling changes the resolution, each decoder-time confidence value is aligned with the center index of its corresponding temporal sampling bin before F2A

injection. The posterior tensor remains attached to the automatic-differentiation graph, and no stop-gradient operation is applied to either the posterior or the resulting confidence bias.

S2. Viterbi Decoding and Structured Objective

Viterbi decoding replaces each log-sum-exp in the forward recurrence with maximization. The implementation stores the best predecessor class and duration for every (t, k) pair and backtracks from $\arg\max_k \delta_{T'}(k)$. The resulting path is optimal only within the configured duration bound and transition support.

For a ground-truth segment sequence S_{GT}, the structured negative log-likelihood is

$$\mathcal{L}_{\mathrm{struct}} = \log \mathcal{Z}(X) - \mathcal{F}(X, S_{\mathrm{GT}}), \tag{S6}$$

and the complete objective is $\mathcal{L}_{\mathrm{struct}} + \lambda_{\mathrm{aux}}\mathcal{L}_{\mathrm{frame}}$. The exact model assigns $-\infty$ to unsupported transitions; finite-precision computation uses $-C$ as its numerical surrogate, with $C = 10^4$.

S2.1. Training and Inference Order

For each training mini-batch, BiasFormer executes the following sequence:

1. The frame branch produces frame features and frame-level logits, and log-softmax normalization converts the logits into emission scores.
2. Log-domain forward–backward inference combines the emission, duration, and transition potentials to compute frame marginals $\gamma_{t,k}$ and confidence biases b_t.
3. Each b_t is added to the corresponding F2A attention logits before the softmax, so action-token queries attend to reliability-reweighted frame keys and values.
4. USMH evaluates the output score sequence with the segmental CRF. The structured negative log-likelihood is added to the auxiliary frame-level classification loss.
5. A single backward pass updates the backbone, F2A module, and structured-model parameters. The posterior is not detached, and no EM-style alternating update is used.

At inference time, the frame-logit, posterior, confidence-bias, and F2A steps remain unchanged. Loss evaluation and backpropagation are omitted, and max-sum Viterbi decoding in USMH returns the highest-scoring segment sequence within the configured duration bound and transition support.

S3. Transition-Support Construction

The reported masks are deterministic functions of the standard model class indices $0, \ldots, K-1$. They are fixed once for each dataset and reused without change across splits. They are not estimated from annotated transition frequencies, and their construction does not use validation/test labels, task identity, or transcripts. The index order is the dataset's standard class mapping with no additional task-stage remapping; consequently, these supports are index-order regularizers rather than exact semantic task grammars. Table S1 records the dataset-specific choices.

S3.1. Free Support

For K classes, set $M_{ij} = 1$ for every ordered pair (i, j). Self-loops are retained. The transition scores U_{ij} remain learnable.

S3.2. Order-Constrained Support

For `monotone_skip` with maximum jump J,

$$M_{ij}^{\mathrm{ms}} = \mathbb{I}(0 < j - i \leq J). \tag{S7}$$

This topology excludes self-transitions and backward-index transitions and bounds a forward jump by J. For `upper`,

$$M_{ij}^{\mathrm{up}} = \mathbb{I}(j \geq i), \tag{S8}$$

which permits latent segment-level self-transitions and arbitrary forward jumps while excluding backward-index transitions. Ground-truth frame labels are merged into maximal same-class segments before structured scoring, but the `upper` candidate support retains self-transitions. Consequently, $D_{\max}$ bounds each latent segment under this support, while adjacent same-class latent segments may collapse into a longer contiguous frame-level action run after decoding. Unsupported entries have theoretical score $-\infty$ and receive the numerical surrogate $-C$ in finite-precision computation. The transition scores on supported entries remain learnable.

Table S1. Transition-support configurations and provenance used for the reported main results. "Standard class index" denotes the index supplied by the dataset mapping used by the model; no additional stage remapping is applied.

Dataset	Mask mode	J	Index convention	Additional annotation or side information used
Breakfast	`monotone_skip`	3	Standard class index; fixed across splits	None
GTEA	`upper`	N/A	Standard class index; fixed across splits	None
EgoProceL	`monotone_skip`	2	Standard class index; fixed across splits	None
EPIC-KITCHENS	`monotone_skip`	2	Standard class index; fixed across splits	None

S4. Split-Specific Reproducibility Record

Table S2 summarizes the executed configurations and evaluation artifacts used for the reported results.

Table S2. Configuration fields required for reproduction.

Field	Verified value or artifact
Feature source and preprocessing	Pre-extracted I3D features from the official or FACT-released resources, or generated with the FACT-provided processing pipeline; BiasFormer and FACT use identical resulting feature inputs
Split source and unit	Official participant-based splits for Breakfast and GTEA; FACT-released 80/20 complete-video split for EgoProceL; official EK-100 video-level split for EPIC-KITCHENS
Split independence scope	Disjoint complete-video lists for all datasets; participant-disjoint for Breakfast and GTEA; participant independence not assumed for EgoProceL; official mixed seen/unseen-participant evaluation for EPIC-KITCHENS
Label mapping and background handling	Unmodified dataset-specific label mappings and background-class handling from the FACT evaluation pipeline
Temporal sampling and output upsampling	Sample rates 1 (Breakfast/GTEA), 3 (EgoProceL), and 4 (EPIC-KITCHENS); sampled predictions are restored to the original sequence length using the unmodified FACT routine before evaluation
Backbone initialization	FACT backbone initialized randomly and trained from scratch; action queries sampled from $\mathcal{N}(0,1)$; other neural layers use PyTorch defaults
U_{ij}, duration, and emission initialization	Transition residual scores and emission-state biases initialized to zero; normal duration with $(\mu_k, \sigma_k) = (20, 10)$ for Breakfast/GTEA; tabular duration with logits initialized to zero for EgoProceL/EPIC-KITCHENS
Posterior detach/gradient behavior	Posterior tensors are not detached; gradients propagate through log-domain forward–backward in a single backward pass
Random seeds and run counts	Seeds 1, 42, and 1337 for Breakfast and GTEA; one training run for EgoProceL and EPIC-KITCHENS
Epochs and batch size	100/2 (Breakfast), 400/1 (GTEA), 350/1 (EgoProceL), and 200/1 (EPIC-KITCHENS), reported as epochs/batch size
Optimizer and scheduler	Adam, learning rate 10^{-4}, weight decay 0; step decay at epoch 50 (Breakfast) and epoch 250 (GTEA/EgoProceL), disabled for EPIC-KITCHENS; no warm-up
Dropout, channel masking, and clipping	Backbone dropout 0.0 except 0.2 on GTEA; USMH emission-head dropout 0.0 for all datasets; channel masking 0.3 except 0.5 on GTEA; gradient clipping at 10.0
Loss weights and fixed constants	$\lambda_{\text{aux}} = 0.2$; $\lambda_{\text{attn}} = 0.2$ except 1.0 on GTEA; $C = 10^4$; $\epsilon = 10^{-8}$; $\sigma_{\min} = 10^{-3}$
Mixed precision and gradient accumulation	Full FP32; no mixed precision or gradient accumulation; one optimizer update per mini-batch
Checkpoint selection and early stopping	Checkpoint with the minimum total training loss in each fixed-length run; no early stopping or validation/test-based checkpoint selection
$D_{\max}$ unit and value	Decoder time steps; 60 for Breakfast, 200 for GTEA, and 32 for EgoProceL/EPIC-KITCHENS
Transition-support artifact	Deterministic formulas and dataset settings in Section S3; no split-specific artifact
Evaluation-code source	Unmodified public evaluation pipeline from the FACT release accompanying Lu and Elhamifar (2024)

S5. Additional Analyses

S5.1. Analytical Computational Cost

The FACT paper reports 1.9 million trainable parameters and 5.5 inference GFLOPs for a five-minute video under its comparison protocol. These are literature-reported values rather than local measurements. BiasFormer reuses the FACT backbone. Its additional parameters comprise transition scores, K emission-state biases, and either $2K$ normal-duration parameters or $KD_{\max}$ tabular-duration logits. A dense transition-score allocation therefore gives the upper bounds

$$\Delta P_{\text{normal}} = K^2 + 3K, \qquad \Delta P_{\text{tabular}} = K^2 + K + KD_{\max}.$$

For GTEA, $K = 11$ and the normal duration model is used, so the parameter increment is at most 154, below 0.01% of the rounded 1.9-million-parameter FACT reference. The `upper` support contains $K(K+1)/2 = 66$ edges; with $D_{\max} = 200$, the additional dynamic-programming computation is $\mathcal{O}(T' \times 200 \times 66)$ and the streamed DP-state storage is $\mathcal{O}(11T')$. These analytical terms are not converted into estimated latency or GPU memory.

S5.2. Sensitivity Analysis

The sensitivity analysis uses GTEA split 1 and seed 1337. It varies one factor at a time while fixing the other two factors at the main configuration, $\lambda_{\text{attn}} = 1.0$, $D_{\max} = 200$, and `upper`. The tested values are $\lambda_{\text{attn}} \in \{0, 0.5, 1.0, 2.0\}$, $D_{\max} \in \{100, 200, 300\}$, and `free`, `monotone_skip` ($J = 2$), and `upper` transition supports. Every run has a fixed training length, and the checkpoint with the minimum total training loss is evaluated. The main manuscript reports F1@50, Edit, and Acc for every setting. The sensitivity runs were not used to revise the main configurations.
